# Starvar: symptom-based tool for automatic ranking of variants using evidence from literature and genomes

**DOI:** 10.1186/s12859-023-05406-w

**Published:** 2023-07-21

**Authors:** Șenay Kafkas, Marwa Abdelhakim, Mahmut Uludag, Azza Althagafi, Malak Alghamdi, Robert Hoehndorf

**Affiliations:** 1 Computational Bioscience Research Center, King Abdullah University of Science and Technology, 23955 Thuwal, Saudi Arabia; 2 Computer, Electrical and Mathematical Sciences and Engineering Division, King Abdullah University of Science and Technology, 23955 Thuwal, Saudi Arabia; 3 Computer Science Department, College of Computers and Information Technology, Taif University, 21655 Taif, Saudi Arabia; 4 Medical Genetic Division, Department of Pediatrics, College of Medicine, King Saud University, 2925 Riyadh, Saudi Arabia

**Keywords:** Variant ranking, Variant prioritization, Text mining, Next generation sequencing, Genomics

## Abstract

**Background:**

Identifying variants associated with diseases is a challenging task in medical genetics research. Current studies that prioritize variants within individual genomes generally rely on known variants, evidence from literature and genomes, and patient symptoms and clinical signs. The functionalities of the existing tools, which rank variants based on given patient symptoms and clinical signs, are restricted to the coverage of ontologies such as the Human Phenotype Ontology (HPO). However, most clinicians do not limit themselves to HPO while describing patient symptoms/signs and their associated variants/genes. There is thus a need for an automated tool that can prioritize variants based on freely expressed patient symptoms and clinical signs.

**Results:**

STARVar is a Symptom-based Tool for Automatic Ranking of Variants using evidence from literature and genomes. STARVar uses patient symptoms and clinical signs, either linked to HPO or expressed in free text format. It returns a ranked list of variants based on a combined score from two classifiers utilizing evidence from genomics and literature. STARVar improves over related tools on a set of synthetic patients. In addition, we demonstrated its distinct contribution to the domain on another synthetic dataset covering publicly available clinical genotype–phenotype associations by using symptoms and clinical signs expressed in free text format.

**Conclusions:**

STARVar stands as a unique and efficient tool that has the advantage of ranking variants with flexibly expressed patient symptoms in free-form text. Therefore, STARVar can be easily integrated into bioinformatics workflows designed to analyze disease-associated genomes.

**Availability:**

STARVar is freely available from https://github.com/bio-ontology-research-group/STARVar.

**Supplementary Information:**

The online version contains supplementary material available at 10.1186/s12859-023-05406-w.

## Background

In the European Union, a condition is defined as rare if it affects fewer than 1 in 2000 people [1]. There are many different rare diseases. Although each of these diseases is rare individually, collectively, they represent a significant component of overall morbidity. Rare diseases contribute to mortality worldwide with estimates ranging from 3.5 to 5.9% of the world’s population [2]. The majority are thought to be caused by inherited genetic variants or occur randomly (*de novo*) in a person who is the first to express phenotypes in an otherwise healthy family. Significant improvements and cost reduction in next-generation sequencing (NGS) technologies, especially over the last decade, have enabled scientists to use them increasingly to study and diagnose rare diseases. Exome and genome sequencing reveals thousands to millions of genetic variants in a typical individual. The main challenge is to identify the tiny subset of variants (typically one or a few) that cause a disease’s phenotype.

While many software methods and tools prioritize variants based on biochemical and genomic features such as conserved regions, allele segregation, and population frequency characteristics, only a few of them, including Exomiser [3] and DeepPVP [4], take into account patient phenotypes and their similarities to phenotypes observed in genetic animal models as well as evidence from the literature. For example, to predict the impact of a missense variant SIFT [5] uses the degree of protein sequence conservation and PolyPhen-2 [6] uses protein sequence and structure. On the other hand, DeepPVP [4] and Exomiser [3] are examples of phenotype-driven methods utilizing semantic similarity between the known gene–phenotypes and patient–phenotypes, while AMELIE [7] prioritizes candidate disease associated genes by utilizing the patient’s genotype as well as the phenotypic similarity between her/his phenotypes and the phenotypes mentioned in the primary literature.

Ontologies are used in the biomedical domain to analyze relations between the bio-entities, such as genotype–phenotype and environment–phenotype relations. Because the ontologies are open and interoperable resources describing concepts such as human phenotypes and diseases and gene function, existing ontology-based variant prioritization tools rely on information in the form of concept identifiers (IDs). For example, Exomiser accepts phenotypes in the form of HPO codes and diseases in the form of OMIM codes as input. However, as research areas evolve over time, ontologies are inherently incomplete [8] and will not cover all the concepts described in the literature. For these cases, ontology-based variant prioritization tools cannot be used accurately. For example, “retinal ischemia”, entirely missing in HPO, returns 1775 hits in PubMed (search performed on 24 February 2022 and restricted to Titles/Abstracts). Although, several related phenotypes can be found in HPO, including “retinal arterial occlusion” (HP:0025326) and “retinal vein occlusion” (HP:0012636), and “tissue ischemia” (HP:0033401); “retinal ischemia” cannot be coded with any of these concepts. The inability of ontology-driven tools to use arbitrary terms to prioritize variants means they cannot benefit from the full potential of existing evidence from the literature. To fill this gap, we propose a new method, STARVar, which relies on symptoms and clinical signs—both in the form of HPO codes and as flexibly expressed terms—and prioritizes variants by combining a classifier using evidence from the biomedical literature and another using evidence from genomes.

## Implementation

### Dataset used to develop and test the literature evidence-based classifier

To develop the literature evidence-based classifier, we generated a training and test dataset by using the known variants from ClinVar (release: Apr-04-2021) [9] and their known phenotypes from the HPO database [10], where the phenotypes are mapped to their HPO codes [11]. For the Protein–Protein Interaction (PPI) data, we used a total number of 841,068 pairs from STRING [12] with high confidence only (STRING score$>=$700). We pre-processed the literature to calculate the association strength by using Jaccard similarity coefficient between the publication records of a set of symptoms and a variant, its associated gene, and PPIs. We collected literature evidence about the variants from 32,923,095 PubMed abstracts [13] (downloaded on Dec-15-2021). We indexed the abstracts using Elasticsearch [14] for variant and gene mentions. We used 57,730,618 gene–abstract pairs [15] and 3,590,328 variant–abstract [16] pairs provided by a widely used resource, PubTator [17]. In addition, we gathered and used a total number of 1,419,508 known variant–abstract pairs from ClinVar.

To generate the training and test dataset, we selected the positive samples from the pathogenic and likely pathogenic variants that are present in ClinVar and associated with phenotypes in the HPO database [10] and linked to a disease in OMIM [18]. We obtained a total number of 89,203 pathogenic or likely pathogenic variants with their phenotypes linked to HPO and associated genes linked to HUGO Gene Nomenclature Committee (HGNC). We selected the negative samples among the benign variants of the same genes as the pathogenic/likely pathogenic variants. Therefore, we aim to allow the model to learn to identify the harmful variants even if its associated gene presents another variant that is benign. Benign variants in ClinVar are identified based on the ACMG Standards and Guidelines [19]. The standards classify a variant with high confidence as benign if its minor allele frequency (MAF) are too high for the disorder, its observation in controls is inconsistent with the disease penetrance, if the well-established functional studies show no deleterious effect, or if it is not segregated with the disease (i.e. in the affected individuals). We obtained a total number of 267,779 benign variants (regardless of the link to phenotypes through OMIM) from ClinVar. We randomly sampled an equal number of positive ( 90K) and negative ( 90K) samples from these variants. Among those, we randomly selected 70% of the samples to train the classifier and the remaining 20% and 10% are used for validation and testing. During the feature generation, any missing values are imputed with zero.

### Generation of synthetic patient datasets

We generated two datasets of synthetic patients to evaluate STARVar. The first dataset (PAVS-synthetic dataset) covers clinically validated variants from an in-house database containing variants observed in Saudi individuals, the Phenotype-Associated Variants in Saudi Arabia (PAVS) [20]. PAVS is a database that combines a set of clinically validated pathogenic variants with a set of manually curated pathogenic variants observed in the Saudi population and their associated phenotypes. These phenotypes are mapped to their HPO codes. Therefore, this dataset was used as a benchmark to compare STARVar against the other phenotype-based variant prioritization tools that can only work with HPO-codes. The second dataset (GPCards-synthetic dataset) covers the variants from a public, manually curated database of genotype–phenotype associations, called GPCards [21]. The phenotypes in GPCards are expressed as terms without relying on a structured vocabulary or ontology. Therefore, we used this dataset to demonstrate the novel and distinct contribution of STARVar.

**Fig. 1:**
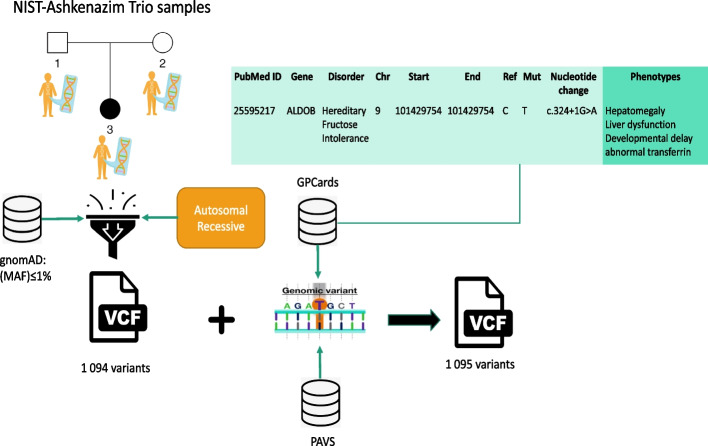
Synthetic patient generation Synthetic patients are generated by using the NIST-Ashkenazi Trio samples, filtered based on the autosomal recessive mode of inheritance and MAF values. We add the reported disease-associated variants either from GPCards (a public database covering manually curated genotype-phenotype associations) or PAVS (a database that covering clinically validated pathogenic variants and their associated phenotypes observed in the Saudi population) to the VCF file to form the synthetic patients

Figure 1 depicts our synthetic patient generation process. We used the NIST-Ashkenazim Trio samples [22] to provide the sequence data of the synthetic patients and the corresponding curated genotype–phenotype associations for the variants associated with the diseases. We first filtered the autosomal recessive variants of the affected son. Then, we selected the rare variants, with Minor Allele Frequency (MAF)$\le$1% in all of the populations from gnomAD [23], resulting in 1,094 variants.

We selected 136 clinically validated homozygous variants from PAVS which do not exist in Clinvar (release: Apr-04-2021) and randomly 50 homozygous variants from GPCards and combined them with the 1,094 filtered variants from the NIST-Ashkenazim Trio samples to generate synthetic patients for the datasets. Both PAVS and GP-Cards contain variants other than homozygous. As we filtered the NIST sample’s variants based on the autosomal recessive mode of inheritance, we selected only the homozygous variants from these datasets. Preparation of the GPCards-synthetic dataset involved some semi-automated work. In particular, we identified the genomic locations of the variants provided with their HGVS codes [24] based on genome assembly GRCh38 by using TransVar [25]. We also represented the phenotypes in a common format, as this was not the case in GPCards. Therefore, we limited this dataset to 50 patients, which is a size that we were able to manage to handle semi-automatically. Both synthetic datasets are available from https://github.com/bio-ontology-research-group/STARVar.

### Variant annotation

We used VEP [26] to annotate variants with an extensive set of information, including the rsIDs of variants (Reference SNP ID assigned by dbSNP), their genomic locations and associated gene symbols (from HGNC), canonical transcript availability, variant consequences [27], SIFT deleteriousness score, PolyPhen-2 pathogenicity score, PPIs from STRING, genotype–phenotype annotations from PAVS. SIFT [5] uses sequence similarity from multiple sequence alignments to predict whether an amino acid substitution affects protein function. PolyPhen-2 (Polymorphism Phenotyping V2)[6] predicts the probability of a missense mutation being damaging by using a naïve Bayesian approach. To determine the variant consequence, VEP first maps each variant to the reference genome and then identifies all overlapping Ensembl [28] transcripts. It then applies a rule-based approach to predict each allele’s effects on each transcript. The complete set of variant consequences (36 in total) ordered by their severity is available from VEP’s website [27]. Ensembl identifies a single representative transcript as canonical for every locus [29]. Hierarchically, it looks for: (1) the longest Coding Sequence (CDS) translation with no stop codons, (2) the longest Ensembl/Havana merged translation with no stop codons, (3) the longest translation with no stop codons, and if there is no translation, (4) the longest non-protein-coding transcript. STARVar operates only on those canonical transcripts. PPIs are not covered by the VEP’s default annotations. Therefore, we gathered PPIs from STRING and generated a custom dataset to use with the VEP workflow. Similarly, we generated custom data for our in-house PAVS dataset. We made our VEP annotation workflow, along with the custom datasets, publicly available via GitHub, providing an automated annotation of any VCF file for the known variants included in PAVS.

### Processing patient samples

We tested STARVar on a family presented at King Saud University Hospital (KSU) in Riyadh. The causative variant of the affected individual is not included in PAVS.

We constructed DNA libraries with QIAGEN QIAseq FX DNA Library kit. We sequenced each individual on Illumina NovaSeq6000 with a 30X coverage. We used the bcbio-nextgen tool kit [30] to align the genomes to the GRCh38 human reference genome and to call variants (we used GATK Haplotype caller). After the variant calling, we first filtered the variants for a MAF <1% by using Slivar [31] and retained the autosomal recessive ones.

We then annotated the remaining variants by using VEP, including variant rsID, gene name, canonical transcript availability, variant consequence, PPIs from STRING, SIFT deleteriousness score, PolyPhen-2 pathogenicity score, and genotype–phenotype annotations of the Saudi population from PAVS. Finally, we used the patient’s clinical signs and symptoms observed and reported by the clinician and ranked the canonical variants with STARVar. We used the phenotypes in the free text form and variant consequences as genomics evidence in STARVar.

## Results

### STARVar combines literature and genomics evidence to rank variants

**Fig. 2:**
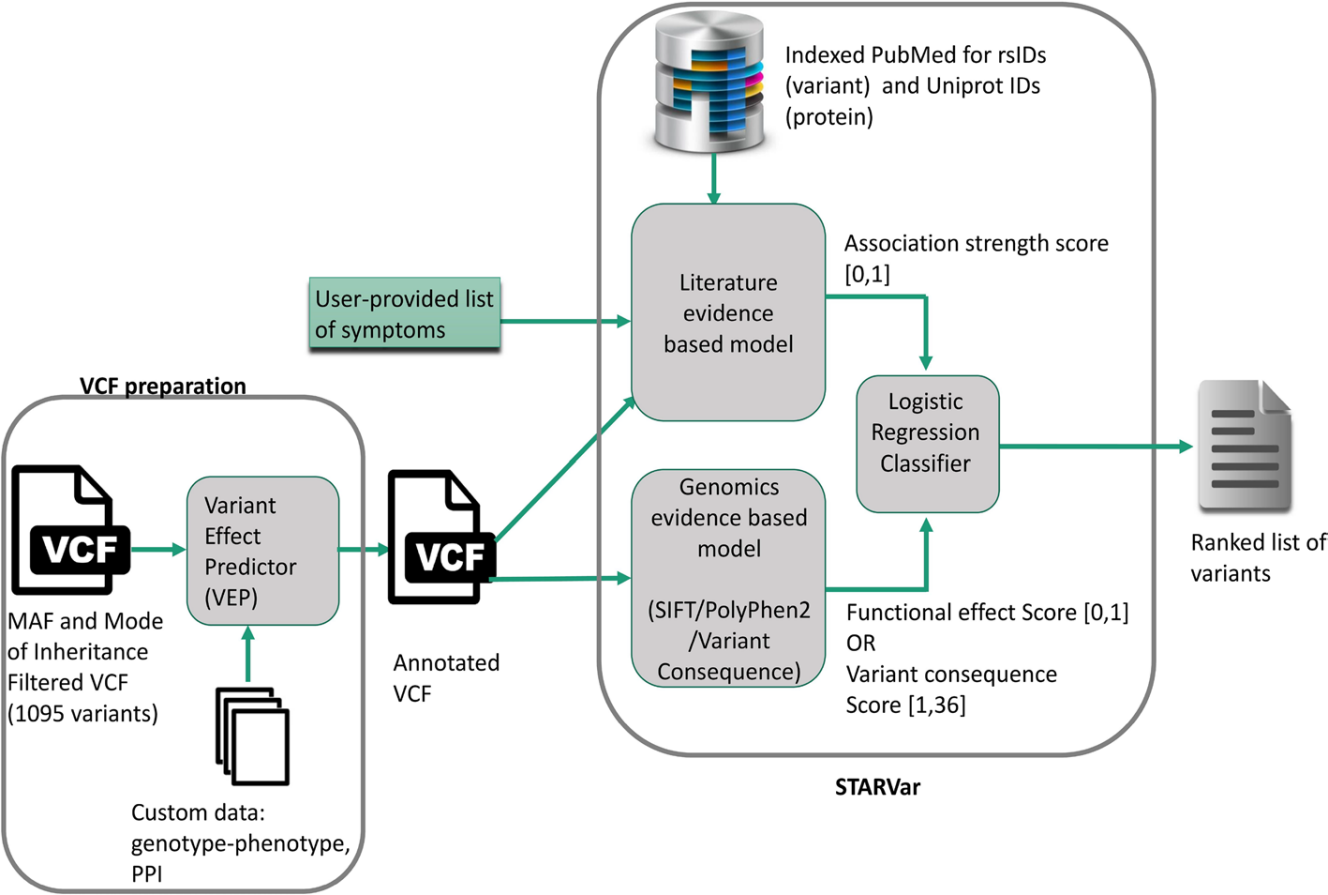
STARVar System Overview. STARVar takes a VCF file annotated by VEP and a list of patient symptoms and combines predictions from two different classifiers to rank the variants

We developed STARVar as a novel tool that takes as input a VCF file annotated by VEP, ideally filtered for the candidate variants, and a list of patient symptoms (either as HPO codes or as arbitrary terms) and combines predictions from two different classifiers to prioritize the variants. Figure 2 depicts the overall system overview. One of the classifiers uses evidence from the literature to measure the association strength between a variant and a list of patient symptoms. The other classifier relies on genomics evidence. STARVar provides three options as genomics evidence; SIFT, Polyphen-2, and Variant Consequence (see Materials and Methods).

We trained another classifier by using the logistic regression algorithm to combine the evidence from the literature and genetic evidence. We used the scikit-learn python package to developed the logistic regression classifiers [32]. We trained all the models by using the default parameters from this package (Inverse of regularization strength set to 1, penalty as L2 norm, and solver is L-BFGS). To train the literature evidence-based classifier, we used three features ($F_{1}$, $F_{2}$, and $F_{3}$) representing the literature-based association strengths between a given set of patient symptoms and a variant, its associated gene, and PPIs. We used Jaccard indices to measure the association strengths as follows:

Let $t = \{t_{1}$,$t_{2}$, $t_{3}$, ...,$t_{N}\}$ represent a set of phenotype terms (either as HPO codes or as text). $S_{x}$ denotes the set of articles containing *x*, *x* being a phenotype, a variant, or a gene.

The association strength between a variant *v* and a set of terms *t* is calculated based on Jaccard index as follows:

$$\begin{aligned} F_{1}=\frac{\sum _{i=1}^{N}\frac{S_{v} \cap S_{t_{i}}}{S_{v} \cup S_{t_{i}}}}{N} \end{aligned}$$

The association strength between a gene *g* and a set of terms *t* is calculated based on Jaccard index as follows:

$$\begin{aligned} F_{2}=\frac{\sum _{i=1}^{N}\frac{S_{g} \cap S_{t_{i}}}{S_{g} \cup S_{t_{i}}}}{N} \end{aligned}$$

The association strength between PPIs of a gene *g* {$g_{1}$,$g_{2}$, $g_{3}$, ...,$g_{M}$} and a set of terms *t* is calculated as follows:

$$\begin{aligned} F_{3}=\frac{\sum _{j=1}^{M}\sum _{i=1}^{N}\frac{S_{g_{j}} \cap S_{t_{i}}}{S_{g_{j}} \cup S_{t_{i}}}}{N.M} \end{aligned}$$

We stored PubMed IDs of abstracts relevant to the known variants, genes, and HPO codes. If a patient’s phenotype is provided as an HPO ID, STARVar looks up for stored PubMed abstracts IDs to calculate the Jaccard Indices. If a symptom is provided directly as a term, STARVar finds the relevant PubMed IDs by searching the indexed titles and abstracts for the provided symptom as a phrase (searching the complete expression within the quotes), then dynamically downloads the relevant PubMed IDs and calculates Jaccard Indices accordingly.

**Fig. 3:**
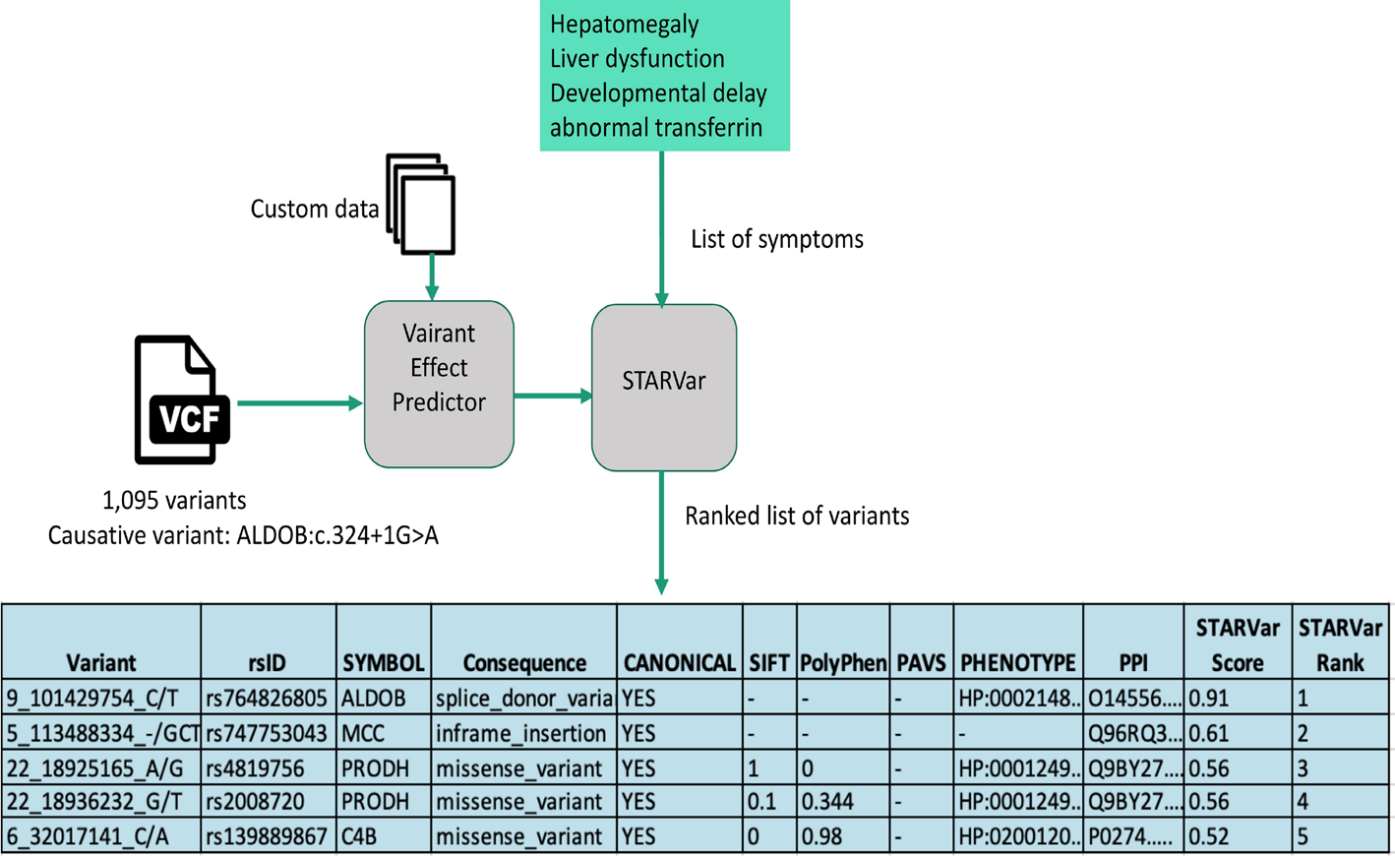
Variant ranking with STARVar. This figure depicts STARVar’s ranking process on a sample, synthetic patient having 1095 variants and a causative variant in ALDOB. STARVar runs on the VCF file covering variants annotated with the custom data by using VEP and utilises user-provided symptom list to rank the variants

STARVar obtains the final prediction scores by combining the individual prediction scores from the literature evidence-based classifier and the genomics evidence-based classifier. The literature evidence-based classifier, which relies on the logistic regression algorithm, assigns prediction scores between 0 and 1 to a variant. For the variant consequences genomics evidence, we assigned weights to the consequence terms between 1 and 36 based on their severity defined by VEP [27]. In the case that SIFT/Polyphen-2 is used as the genomics evidence, the classifier assigns a score between 0 and 1. To combine the evidence scores from the two classifiers, STARVar uses another logistic regression based classifier that utilise the scores from the two classifiers. The final prediction scores from this classifier are used to rank variants. Figure 3 depicts STARVar’s variant ranking process and results on a synthetic patient having the ALDOB:c.324+1 G>A variant from the GPCards-synthetic dataset with clinically observed symptoms covering “Hepatomegaly”, “Liver dysfunction”, “Developmental delay”, “Abnormal transferrin”. STARVar ranks this particular disease-associated variant at the top when variant consequence is used as genomics evidence.

### Evaluation of STARVar on the test dataset

The feature coefficients of the logistic regression-based classifier were 2.32, 51.96 and 40.31 for the literature-derived features $F_{1}$, $F_{2}$, and $F_{3}$, respectively. The result shows that the classifier assigns more weight to the genes ($F_{2}$) than to the PPIs ($F_{3}$) and the variants ($F_{1}$).

The literature evidence-based classifier achieved an accuracy (i.e., the overall proportion of true predictions, positive and negative) of 0.75 on the test set.

Table 1 presents the results on the test set in terms of precision, recall and F-score for positive and negative classes separately. The classifier achieved respectively F-scores of 0.79 and 0.68 on the negative and positive variants.

**Table 1:** Decoupled logistic regression classifier’s performance evaluation on the test set (ClinVar)

Classifier	Class	Precision	Recall	F-score
Literature	Negative	0.66	0.99	0.79
Literature	Positive	0.98	0.52	0.68
Literature and variant consequence	Negative	0.92	0.99	0.95
Literature and variant consequence	Positive	0.99	0.92	0.95

The feature coefficients of the logistic regression based classifier were 0.24 and 40.47 for the literature-based classifier and the genetic features (Variant Consequence) respectively. The results show that the classifier assigns more weight to the genetic feature. The classifier achieved an accuracy of 0.95 on the test set and an F-score of 0.95 on both, negative and positive variants (Table 1).

### Performance comparison on synthetic patients

We compared the performance of STARVar against several other phenotype-based variant prioritization tools on the PAVS-synthetic dataset that comprises phenotypes mapped to HPO. Table 2 shows the results for the disease-associated variants ranked at the top and within the top 5 and 10. STARVar achieved its best performance when we used Variant consequence from VEP as genomics evidence. It ranked the disease-associated variant at the top for 66 out of 136 patients and within the top 5 and top 10 for 120 and 132 out of 136, respectively. STARVar’s literature evidence-based classifier ranked the disease-associated variant at the top for 30 out of 136 patients and within the top 5 and top 10 for 44 and 50 out of 136, respectively. The genomic evidence-based classifier ranked the disease-associated variant at the top for 55, 49 and 32 patients and within the top 5 and top 10 for 100 and 132 of them when used with SIFT, PolyPhen-2 and Variant Consequence. STARVar ranked the disease-associated variant at the top in 49 and 43 and within the top 10 for 107 and 84 cases when used with PolyPhen-2 and SIFT. We compared STARVar’s performance against Exomiser [3] and AMELIE [7], two well-known phenotype-based variant prioritization tools. Exomiser ranked the disease-associated variant at the top for 76 patients and within the top 5 and 10 for 90 and 96 of them, respectively. AMELIE ranked the disease-associated variant at the top for only 34 patients but within to top 5 and top 10 in 83 and 101 cases, respectively. Altogether, results show that Exomiser narrowly performs better than STARVar with Variant Consequences from VEP for ranking the disease-associated variant at the top. However, STARVar outperformed all the other classifiers for ranking the disease-associated variant within the top 5 and top 10.

**Table 2:** Performance comparison on the PAVS-synthetic dataset

Classifier	Hits@1	Hits@5	Hits@10
STARVar (Literature+Variant Consequences)	66	120	132
STARVar (Literature+SIFT)	43	76	84
STARVar (Literature+ PolyPhen-2)	49	98	107
STARVar (Literature)	30	44	50
STARVar (Variant Consequence)	32	32	132
SIFT	55	100	100
PolyPhen-2	49	100	100
AMELIE	34	83	101
Exomiser	76	90	96

### Performance evaluation on synthetic patients with textual phenotypes

We evaluated STARVar on the GPCards-synthetic dataset using their phenotypes which are listed as terms without reference to a controlled vocabulary. As shown in Table 3, STARVar achieves the best performance when Variant Consequence is used as genomics evidence. It ranked 38 out of 50 disease-associated variants at the top against 24 and 21 when Polyphen-2 and Sift are used as genomics evidence. Furthermore, it ranked 48 out of 50 disease-associated variants within the top 10 when Variant Consequence is used as genomics evidence.

**Table 3:** Evaluation of STARVar on the GPCards-synthetic dataset

Classifier	Hits@1	Hits@5	Hits@10
STARVar (Literature+Variant Consequences)	38	46	48
STARVar (Literature+SIFT)	21	37	41
STARVar (Literature+ PolyPhen-2)	24	33	40

### Ablation study of the literature-based features

An ablation study helps to study the performance of a machine learning-based system by eliminating certain input components to understand which components are significant to the output. We conducted an ablation study on the literature-based features; $F_{1}$ (Jaccard index of a variant and a patient symptom list), $F_{2}$ (Jaccard index of a gene and a patient symptom list), and $F_{3}$ (Jaccard index of a set of PPIs and a patient symptom list). We evaluated performances on the PAVS-synthetic dataset (see Table 4). Using $F_{1}$, the classifier ranked the disease-associated variant at the top for 34 patients and in the top 10 for 51 patients. Using $F_{2}$, the classifier ranked the disease-associated variant at the top for 48 patients and within the top 10 for 74 of them. Using $F_{3}$, the classifier ranked the disease-associated variant at the top for 12 patients and within the top 10 for 26. Our ablation study shows that $F_{2}$ is the most significantly contributing feature to the classifier’s performance.

**Table 4:** Feature ablation results on the PAVS-synthetic dataset

Feature	Hits@1	Hits@5	Hits@10
$F_{1}$	34	51	51
$F_{2}$	48	65	74
$F_{3}$	12	21	26

$F_{1}$, $F_{2}$ and $F_{3}$ represents Jaccard index of a patient symptom list and a variant, a gene and a set of PPIs respectively

### Analysis of performance based on the gene presentation in literature

Our literature-based classifier relies on the information in the PubMed abstracts. We conducted several experiments to better understand how the number of publications that mention a variant or gene affects STARVar’s performance; we compare with AMELIE, which uses literature information, but do not include Exomiser in this comparison as it does not rely on literature.

**Fig. 4:**
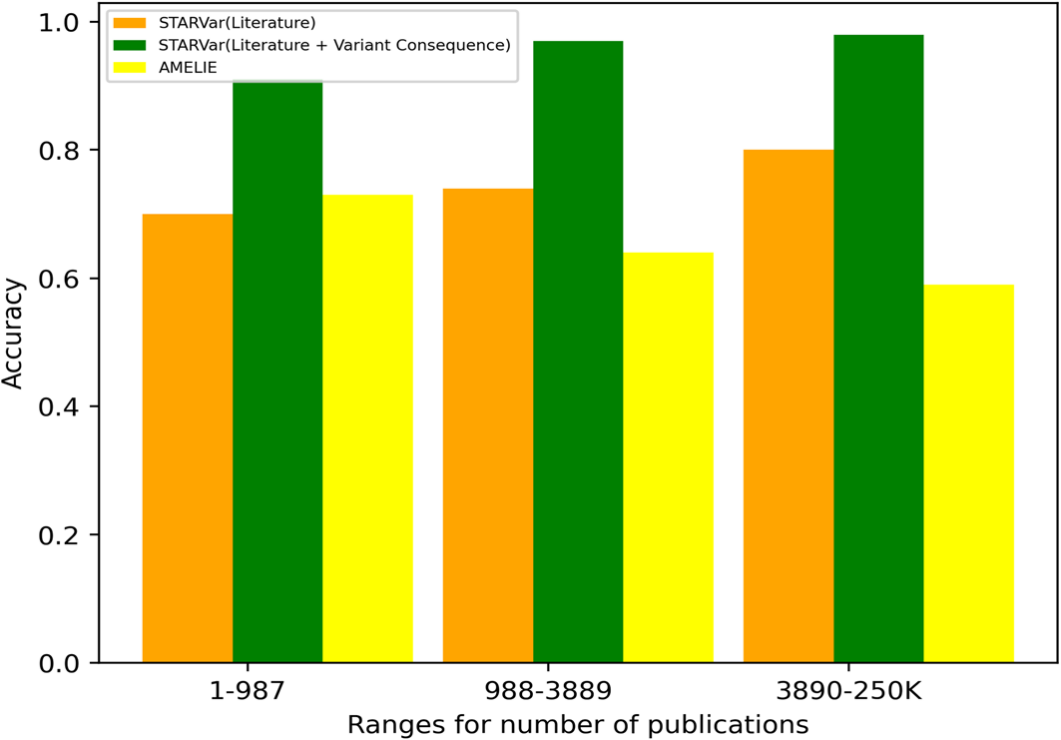
Figure: Performance analysis of AMELIE and STARVar based on the number of publications associated with genes. The x-axis represents the ranges for the number of publications, while the y-axis denotes the accuracy. The graph shows how the accuracy of AMELIE (in yellow) and STARVar varies across different ranges of publication counts, using the literature only in orange and in addition to the variant consequence in green

Figure 4  shows the performance of AMELIE and STARVar on the test set that we gathered from ClinVar (17,841 samples). We generated three bins, each covering almost an equal number of samples (around 5970). Each sample is linked to a number of publications ranging between 1 and  250,000. Our results show that STARVar achieves the lowest accuracy of 0.70 and 0.91 on the genes appearing least frequently in the literature (1–987 publication)] when literature evidence and literature combined with genetic evidence are used. The accuracy improves to 0.74 and 0.97 on the genes having a number of linked publications in the range of (988–3,889) when literature and literature combined with genetic evidence are used. STARVar achieves the highest accuracy of 0.80 and 0.98 on the cases that are well studied and reported in the literature (3890–250,000).

AMELIE’s accuracy, on the other hand, drops from 0.73 to 0.64 and then to 0.59 as the number of publications linked to genes increased. This opposite trend can be explained by the different way in which AMELIE utilizes literature information, i.e., as it ranks articles for a given gene individually and does not rely on the entire literature.

### Application of STARVar to a clinical case

**Fig. 5:**
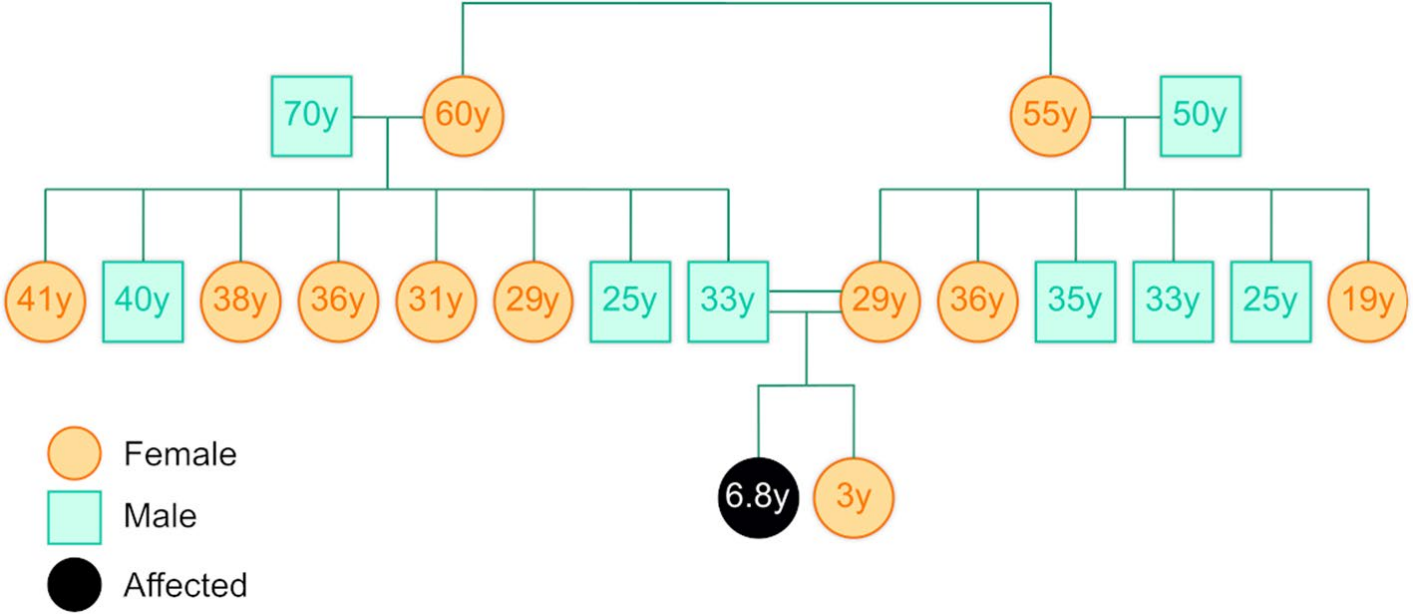
Family Pedigree of the use case with an affected female. This pedigree illustrates the family structure and genetic history of the case study, focusing on an affected female individual (indicated by the shaded circle). Squares represent male individuals, circles represent female individuals, shaded symbols indicate affected individuals, and the numbers represent the ages. Lines connect parents to their children

The consanguineous family is a trio consisting of a mother and a father and their affected child. Figure 5 depicts the family pedigree. The affected child presented to the clinic with “joint contracture”, “subcutaneous nodules”, and “osteolytic lesion on the skeletal survey”. By following the analysis procedure described in the *Processing patient samples* subsection, we obtained a total of 795 variants. We ranked the variants with STARVar and manually analyzed the top-ranking variants. We identified a plausible frameshift variant MMP2:c.1289del p.. Contextual string embeddings for sequence labeling.(Asn430Thrfs*68).

The frameshift introduces a new stop codon downstream of the variant.

The MMP2 gene is linked to the “Multicentric osteolysis, nodulosis, and arthropathy” disease in OMIM (OMIM:259600), a disease that covers the phenotypes observed in the patient. STARVar ranked this variant in the first rank when the variant consequence is used as genomics evidence. The variant segregated well within the family and has been previously confirmed by using Sanger sequencing. It is classified as likely pathogenic (class 2) according to the recommendations of ACMG [19]. Furthermore, the variant had been previously reported as “pathogenic” in ClinVar [33].

However, the frameshift MMP2 variant is the only variant with severe functional consequences among the 795 variants. To make the problem more challenging, we removed the genomic evidence and ranked variants using only literature components of STARVar. STARVar using only literature evidence as input still ranks the MMP2 variant at the first rank. This example illustrates that STARVar can rank variants based on clinical phenotypes, and that the literature component provides information about genotype–phenotype relations, in addition to the genomic evidence.

## Discussion

To the best of our knowledge, STARVar is the first symptom or sign based variant prioritization tool whose functionality is not restricted to phenotypes present in HPO (AMELIE and Exomiser), OMIM (Exomiser), Mammalian Phenotype Ontology (PVP [34], Phevor [35]), Gene Ontology, or Uberon (Phevor). It can operate with a list of phenotypes provided either directly as terms or in the form of HPO codes. Because clinicians do not necessarily express symptoms as HPO codes and HPO does not cover all the possible phenotypes, STARVar offers a unique advantage compared to the other existing phenotype-based tools, being capable of exploiting most of the relevant evidence, particularly from the literature. We compared STARVar’s performance against two well-known phenotype-based variant prioritization tools, AMELIE and Exomiser, on a dataset of clinically validated variants from PAVS. While Exomiser utilizes curated gene–phenotype associations, AMELIE takes into account evidence from the literature during its prediction process. The AMELIE logistic regression-based classifier combines 27 different features to rank all articles in the AMELIE knowledgebase for their ability to explain patient phenotypes. Two of these features are the Phrank [36] phenotype similarity scores of a patient’s phenotypes with the phenotypes mentioned in the article.

Exomiser comprises a set of algorithms for ranking variants by using random-walk analysis of protein interaction networks, clinical relevance, and cross-species phenotype comparisons, in addition to several other computational filters for variant frequency, predicted pathogenicity, and pedigree analysis. Users are required to supply patient phenotypes as HPO codes or OMIM identifiers for Exomiser and HPO codes for AMELIE. Exomiser achieved the best performance for ranking the disease-associated variant at the top, while AMELIE achieved the worst on the 136 individuals selected from PAVS. On the other hand, STARVar outperformed all the other classifiers when ranking the disease-associated variant within the top 10 (see Table 2). As computational tools might not identify the disease-associated variant always at the top, one would consider a wider range of a ranked list while interpreting variants. Therefore, STARVar is advantageous for variant prioritization. We also evaluated STARVar on another set of synthetic patients whose observed phenotypes were expressed textually (GPCards-synthetic dataset). STARVar ranked 48 out of 50 disease-associated variants within the top 10 (and in the first position for 38/50 patients). One could envision converting the symptoms and clinical signs to HPO codes and using one of the existing phenotype-based tools for variant prioritization. Although this would be possible to an extent, as demonstrated by the previous efforts such as ClinPhen [37] and Doc2HPO [38], this would not allow using all the clinical observations. For example, the GPCards-synthetic dataset covered 248 distinct phenotypes in total. There was no exact match in HPO for 83 of the 248 phenotypes. Those 83 phenotypes cover both potential synonyms of existing HPO concepts and new concepts such as “sleep benefit” which we found in 70 relevant articles in PubMed where 4 of these co-mention the phenotype with the disease-associated gene “PINK1” (searched on 13 March 2022). However, STARVar can directly and automatically use those 83 phenotypes.

We further tested STARVar on a single consanguineous family for which clinical and genetic data was available to us. STARVar was able to find the plausible variant in the first position when both literature and genomics evidence was used as well as when considering only literature evidence. However, while this example demonstrates that STARVar works on real patient samples and succeeds in identifying disease-associated variants, a single example is not sufficient to draw general conclusions about how well STARVar works on patient data across different clinical settings, disease types, family pedigrees, or sequencing technologies. While we have made every effort to make our experiments and comparison using synthetic data as realistic as possible, additional clinical samples will need to be analyzed in the future to strengthen the evidence of the utility of STARVar on clinical samples.

STARVar presents limitations due to its dependence on literature, text mining (PubTator), SIFT/PolyPhen-2/VEP, sequencing data as well as test data (PAVS in our case). STARVar obtains final predictions by combining predictions from a literature evidence-based classifier and a genomic evidence-based classifier (PolyPhen-2/SIFT/Variant Consequence). In some specific cases, STARVar may fail to identify the disease-associated variant in the top ranks, especially when there are weak or no signals in the literature regarding the association between a variant and a list of patient symptoms, and no or misleading signals from SIFT, PolyPhen-2, or Variant Consequence regarding the functional effect of a mutation or variant consequences. Weak or no evidence in the literature might be explained by the biomedical literature not guaranteed to contain the complete set of queried patient phenotypes, the phenotypes provided by the user not using the consensus terminology used by authors for describing phenotypes in articles, and PubTator failing to identify some of the mutations or gene names accurately. For example, PAVS contains a disease-associated variant of the CEP152 gene known as NM_014985.3:c.2148-17 G>A and reported with “Encephalopathy” (HP:0001298). STARVar ranks this disease-associated variant at the 86^th^ position when VEP’s variant consequence is used as genomic evidence. Searching “CEP152 AND Encephalopathy ” for the relevant titles or abstracts in PubMed returns zero hits (search performed on 25 April 2022). In addition, VEP identified the variant consequence as “intron variant”, which has a modifier impact (i.e., usually non-coding variants or variants affecting non-coding genes, for which predictions are difficult or there is no evidence of impact). STARVar also fails in the case of ambiguous data in the test data. For example, PAVS contains a disease-associated variant of the COX15 gene (cc.750+85A>G), reported for a patient and responsible for a set of phenotypes including “Amblyopia”, “Bilateral basal ganglia lesions”, “Generalized hypotonia” and “Global developmental delay”. This specific case is annotated as “ambiguous” by the clinician meaning that the variant does not explain the phenotypes. STARVar ranks this disease-associated variant at the 233rd position when VEP’s variant consequence is used as genomic evidence. VEP identified this particular variant’s consequence as “intron variant” and our search in PubMed for the relevant abstracts returns no signal for the variant. More specifically, querying the genes COX15, with the aforementioned phenotypes returned zero hits (search performed on 17 April 2022). Altogether, weak or no signal from the literature or VEP, or ambiguous cases in the test data, lead STARVar to miss some disease-associated variants.

We conducted several experiments to reveal the overall picture on the effect of the number of publications linked to the gene on the STARVar’s performance. We found that, both the literature-based model and the literature plus variant consequence achieve their lowest performance on the genes which least frequently appear in the literature and the performance reaches to the highest on the ones which present highly in the literature. One would expect this behaviour, as STARVar utilise literature evidence for ranking the variants; more articles lead to more reliable evidence from literature and this leads to higher accuracy. Nevertheless, incorporating genetic features helps not only to improve the accuracy but also shrinks the performance gap between the highly presented genes and the less frequently presented ones.

## Conclusion

This study presents STARVar, a tool based on flexibly expressed symptoms and clinical signs for automatically ranking variants in a patient genome. STARVar combines evidence from literature and patient genomes for variant prioritization. To the best of our knowledge, STARVar is the first variant prioritization tool that can use symptoms and clinical signs expressed as arbitrary terms and is not limited to specific controlled vocabularies. STARVar performs similarly to other tools on a synthetic patient-derived dataset but is more general as it can operate with flexibly expressed phenotypes. We demonstrated STARVar’s unique functionality on another synthetic dataset with flexibly expressed patient phenotypes and showed that it could identify 48 out of 50 disease-associated variants within the top 10 hits. STARVar is freely available (see Supplementary Information, Additional File 1) and provides an automated variant prioritization workflow that can be used to analyze research and clinical data.

## Availability and requirements

- Project name: STARVar
- Project home page: https://github.com/bio-ontology-research-group/STARVar
- Operating system(s): Platform independent
- Programming language: Python
- Other requirements: none
- License: 4-clause BSD-style license

## Supplementary Information

**Additional file 1**. This file contains the source code (in python) of STARVar.

## Data Availability

https://github.com/bio-ontology-research-group/STARVar.

## Abbreviations

AMELIE: Automatic Mendelian Literature Evaluation
GO: Gene Ontology
HGNC: HUGO Gene Nomenclature Committee
HPO: Human Phenotypes Ontology
ID: Identifier
KSU: King Saud University
MAF: Minor Allele Frequency
NGS: Next Generation Sequencing
OMIM: Online Mendelian Inheritance in Man
PAVS: Phenotype-Associated Variants in Saudi Arabia
PPI: Protein-Protein Interaction
PVP: PhenomeNet Variant Predictor
SIFT: Sorting Intolerant From Tolerant
STRING: Search Tool for the Retrieval of Interacting Genes/Proteins
STARVar: Symptom-based Tool for Automatic Ranking of Variants
VCF: Variant Call Format
VEP: Variant Effect Predictor
