# Supplementary material for: Starvar: symptom-based tool for automatic ranking of variants using evidence from literature and genomes
Source: BMC Bioinformatics. 2023 Jul 21;24:294. doi: 10.1186/s12859-023-05406-w (PMC10362560; doi:10.1186/s12859-023-05406-w)
Supplement: Supplementary file 1 — Additional file 1. This file contains the source code (in python) of STARVar. [file 12859_2023_5406_MOESM1_ESM.zip › readme.docx]

STARVar:Symptom based Tool for Automatic Ranking of Variants using evidence from literature and genomes

How to use STARVar?

To run STARVar, please follow the steps below:

1. Download the required input files from <https://exrcsdrive.kaust.edu.sa/exrcsdrive/index.php/s/CobjdzXWoWmJbYs> . The password for accessing the data is : 12345
2. An input VCF file containing variants first should be filtered for the candiate variants based on Allele Frequency and/or mode of inheritance. (e.g. synthetic_ALDOB.maf001.AR.vcf) We suggest users to use one of the efficient filtering tools, e.g. slivar for the filtering step. Slivar is available from <https://github.com/brentp/slivar>
3. The filtered VCF file must be annotated by using the Variant Effect Predictor (VEP) tool with the required information (see the VEP_AnnotFields file for the full set of annotation fields by VEP) for analyzing data efficiently. Follow the steps described at <https://github.com/bio-ontology-research-group/pavs_annotation> to install and run the VEP as CWL locally.
4. run STARVar as follows: format: python STARVar.py input.vcf option symptoms

option: STARVar combines scores from the literature based model and genomics evidence scores from either PolyPhen-2, SIFT or Variant Consequence from VEP for variant ranking. Please choose one of them. Use s for SIFT or use p for PolyPhen-2 or use v for Variant Consequence.

symptoms: should be provided as a semi-colon separated single list of symptoms either in free text format or as HPO IDs. For example, "HP:XXXXXX;brain atrophy". Please dont forget to use quotation (") for symptoms.

Example: python STARVar.py gpcards_ALDOB.vep.vcf v "Hepatomegaly;Liver dysfunction;Developmental delay;abnormal transferrin"
